# Identification of glutamine metabolism-related gene signature to predict colorectal cancer prognosis

**DOI:** 10.7150/jca.91687

**Published:** 2024-04-15

**Authors:** Yang Xie, Jun Li, Qing Tao, Yonghui Wu, Zide Liu, Chunyan Zeng, Youxiang Chen

**Affiliations:** 1 Department of Gastroenterology, digestive disease Hospital, The First Affiliated Hospital, Jiangxi Medical College, Nanchang University, Nanchang China.; 2 Jiangxi Clinical Research Center for Gastroenterology, Nanchang, Jiangxi, China.

**Keywords:** Glutamine metabolism-related genes, Colorectal cancer, Prognosis, Survival analysis, Immune cell infiltration.

## Abstract

**Backgrounds:** Colorectal cancer (CRC) is a highly malignant gastrointestinal malignancy with a poor prognosis, which imposes a significant burden on patients and healthcare providers globally. Previous studies have established that genes related to glutamine metabolism play a crucial role in the development of CRC. However, no studies have yet explored the prognostic significance of these genes in CRC.

**Methods:** CRC patient data were downloaded from The Cancer Genome Atlas (TCGA), while glutamine metabolism-related genes were obtained from the Molecular Signatures Database (MSigDB) database. Univariate COX regression analysis and LASSO Cox regression were utilized to identify 15 glutamine metabolism-related genes associated with CRC prognosis. The risk scores were calculated and stratified into high-risk and low-risk groups based on the median risk score. The model's efficacy was assessed using Kaplan-Meier survival analysis and receiver operating characteristic (ROC) curve analysis. Cox regression analysis was employed to determine the risk score as an independent prognostic factor for CRC. Differential immune cell infiltration between the high-risk and low-risk groups was assessed using the ssGSEA method. The clinical applicability of the model was validated by constructing nomograms based on age, gender, clinical staging, and risk scores. Immunohistochemistry (IHC) was used to detect the expression levels of core genes.

**Results:** We identified 15 genes related to glutamine metabolism in CRC: NLGN1, RIMKLB, UCN, CALB1, SYT4, WNT3A, NRCAM, LRFN4, PHGDH, GRM1, CBLN1, NRG1, GLYATL1, CBLN2, and VWC2. Compared to the high-risk group, the low-risk group demonstrated longer overall survival (OS) for CRC. Clinical correlation analysis revealed a positive correlation between the risk score and the clinical stage and TNM stage of CRC. Immune correlation analysis indicated a predominance of Th2 cells in the low-risk group. The nomogram exhibited excellent discriminatory ability for OS in CRC. Immunohistochemistry revealed that the core gene CBLN1 was expressed at a lower level in CRC, while GLYATL1 was expressed at a higher level.

**Conclusions:** In summary, we have successfully identified and comprehensively analyzed a gene signature associated with glutamine metabolism in CRC for the first time. This gene signature consistently and reliably predicts the prognosis of CRC patients, indicating its potential as a metabolic target for individuals with CRC.

## Introduction

Colorectal cancer (CRC) is not only the fourth most common cancer globally, but also the third deadliest, as reported in the latest data in 2020^1^. The incidence and prevalence of CRC have shown a gradual and incremental trend globally from 1999 to 2019, imposing a significant economic burden on global healthcare 2, 3. Geographically, the incidence of colorectal cancer varies, with significantly higher cases observed in economically developed countries like Europe and North America compared to less economically developed countries 4. In China, the overall incidence of CRC has also increased significantly in recent years due to improvements in living standards and changes in dietary patterns 1, 5. Therefore, in light of the high malignancy and poor prognosis associated with CRC, early identification of high-risk factors and the establishment of a prognostic model for CRC can not only help reduce its morbidity and mortality rates, but also guide clinical treatment more effectively.

Glutamine is one of the most abundant amino acids in human plasma and plays various roles, including the regulation of energy metabolism, maintenance of pH homeostasis, and cellular integrity 6-8. Glutamine can be hydrolyzed into glutamate and ammonium ions (NH4+) by glutaminase, while glutamate and ammonia (NH3) can be synthesized into glutamine through the catalysis of glutamine synthetase 8. Several studies have reported significantly lower serum glutamine concentrations in CRC patients compared to the healthy population, possibly due to the increased uptake and utilization of nutrients by cancer cells for proliferation 9-12. Clinical studies have also demonstrated that serum glutamine levels can serve as a prognostic marker in CRC, as glutamine deficiency promotes recurrence and metastasis 13, 14. A meta-analysis finds glutamine significantly improves indicators of humoral and T-cell immune function in patients after radical surgery for colorectal cancer 15. Additionally, glutamine is essential for the treatment of CRC patients, as it can reduce complications and improve treatment outcomes 16. Many genes are closely associated with glutamine metabolism, with glutaminase 1 (GLS1) being an important gene that is up-regulated in CRC patients. Knockdown of the GLS1 gene has been shown to inhibit the proliferation and migration of CRC cells 17. Yu et al. found that mutations in the oncogene Pik3CA lead to glutamine dependence in CRC, suggesting that targeting glutamine metabolism may be an effective treatment for patients with Pik3CA-mutated CRC 18. Qi et al. found that HSF1 stimulated acetyltransferase P300-mediated hyper-enhanced activity, promoted LCC00857 expression, and enhanced SLC1A5-mediated glutamine transport 19. In summary, glutamine metabolism and related genes play a significant role in the development of CRC.

However, previous research has primarily focused on the diagnostic value of glutamine in CRC, with limited studies exploring its prognostic value. Hence, this study aims to develop a predictive model for glutamine and glutamate metabolism-related genes to assess the prognosis of CRC. Such a model will provide valuable insights into identifying potential therapeutic targets for CRC.

## Materials and Methods

### Data Collection and Preprocessing

We obtained the gene expression matrices of the CRC samples from The Cancer Genome Atlas (TCGA) data portal (https://portal.gdc.cancer.gov) as the training set. We used the GSE17536 and GSE103479 datasets as external validation sets. The inclusion criteria for this study were as follows: (1) CRC patient samples with both mRNA sequencing data and survival status; (2) samples with complete clinical information. The TCGA database provided overall survival status for 433 CRC patients, and we also downloaded their clinical information, including survival time, survival status, age, gender, tumor TNM stage, and clinical classification. For further analysis, we selected 433 CRC patients who had complete clinical information. The GSE17536 and GSE103479 datasets contained 177 and 155 CRC patients, respectively, and were normalized using the R package "limma". We obtained genes related to glutamine metabolism from the MsigDB database (https://www.gsea-msigdb.org/gsea/msigdb).

### Construction and validation of prognostic signatures of genes related to glutamine metabolism

We used the "edgeR" package in R software to screen for differentially expressed genes between colorectal cancer and normal tissues. Significance was determined by an adjusted p-value < 0.05 and |log2(FC)| value > 1. We then intersected the downloaded glutamine metabolism-related genes with the differential genes to identify those involved in colorectal cancer development. To identify survival-associated glutamine metabolism genes in colorectal cancer patients, we performed univariate Cox proportional hazard regression analyses to explore the relationship between overall survival (OS) and glutamine metabolism-related genes. Genes with a p-value < 0.05 were considered to be associated with survival in colorectal cancer patients. We employed Least Absolute Shrinkage and Selection Operator (LASSO) regression analysis to minimize overfitting and identify survival-related genes of glutamine metabolism, using TCGA expression profiles. The R package "glmnet" was used to find the best gene models and calculate individualized risk scores using the coefficients. The risk score was calculated using the formula: risk score = gene 1 expression * coefficient + gene 2 expression * coefficient + ... + gene n expression * coefficient 20. Based on the median threshold of the risk score, patients were divided into high-risk and low-risk groups. The R packages "survival" and "survminer" were used to determine the optimal cut-off values of risk scores and plot Kaplan-Meier survival curves. These curves illustrated the difference in OS between the high-risk and low-risk groups, and the area under the curve (AUC) was calculated using the R package "survivalROC" 21 to assess the time-dependent prognostic value of the model 22. Furthermore, we validated the risk signature in the GSE17536 and GSE103479 datasets separately. Clinical characteristics of colorectal cancer patients, such as gender, age, clinical stage, histological grade, and tumor lymph node metastasis (TNM) status, were obtained from the TCGA database. We conducted univariate and multivariate Cox regression analyses to determine whether the model risk scores independently predicted prognoses, considering these clinical characteristics. A p-value < 0.05 was considered statistically significant.

### Nomogram model construction

The construction of a nomogram involved the use of the "rms" and "hmisc" R packages 23. To assess the clinical usefulness of the predictive nomogram, a decision curve analysis was conducted to quantify the net benefits across different threshold probabilities. Additionally, calibration curves were generated to evaluate the agreement level between the nomogram and the ideal observations. ROC curve of the clinical prognostic nomogram for predicting 1-, 2-, and 3-year survival in CRC patients was analyzed by MedCalc 20.0 statistic software.

### Estimation of tumor-infiltrating immune cells between high- and low-risk groups

The ssGSEA algorithm is based on 29 immune genomes, which include genes associated with various immune cell types, functions, pathways, and checkpoints. We utilized the ssGSEA algorithm through the R package, namely GSVA, GSEABase, GSVA, ggpubr, reshape2, and limma, to thoroughly evaluate the immunological profile of each sample in the study 24.

### Drug sensitivity prediction analysis of glutamine metabolism-related genes in CRC

We obtained drug sensitivity data from the CellMiner database (https://discover.nci.nih.gov/cellminer). Subsequently, scatter plots were utilized to display the correlations between the expression of genes associated with glutamine metabolism and the various drug sensitivity scores. These scatter plots were generated using the R/Bioconductor software package, including ggplot2, impute, limma, and ggpubr. The correlations were assessed using Pearson correlation analysis.

### Immunohistochemistry

Tissue slides from 10 colorectal cancer (CRC) patients were obtained from the Department of Pathology, The First Affiliated Hospital of Nanchang University. Immunohistochemical (IHC) staining was performed according to standard laboratory protocols as previously described 25. The GLYATL1 antibody was purchased from Abmart (PHB9276, Shanghai) at a dilution of 1:100, while the CBLN1 antibody was purchased from Abmart (PK30087, Shanghai) at a dilution of 1:100. The GLYATL1 and CBLN1 IHC scores were determined based on the intensity and percentage of staining in tumor cells. Staining intensity was classified as 0 (negative), 1 (weak), 2 (moderate), or 3 (strong), and the percentages were assigned as follows: 1 (0-25%), 2 (26-50%), 3 (51-75%), or 4 (>75%). The total IHC staining scores were calculated as the product of the intensity and percentage. IHC scores less than 6 were defined as the low expression group, while scores greater than 6 were defined as the high expression group. Immunohistochemical staining was independently assessed and examined by two observers (Qing Tao and Yong hui Wu).

### Statistical Analysis

Statistical analyses were conducted to assess the variables in the study. Continuous variables were expressed as mean ± SE, while categorical variables were expressed as frequency (n) and proportion (%). Differences between variables were analyzed using t-tests, non-parametric tests, and chi-square tests. Univariate and multivariate Cox regression analyses were performed to estimate the predictive power of immune-related risk characteristics and clinical characteristics. Kaplan-Meier analyses were used to estimate the overall survival rates of different groups, and log-rank tests were employed to assess the significance of differences between the groups. Statistical analyses were conducted using GraphPad Prism 8.0 and R software version 4.3.1^26^.

## Results

### Identification of prognostic genes associated with glutamine metabolism in CRC

**Figure 1:**
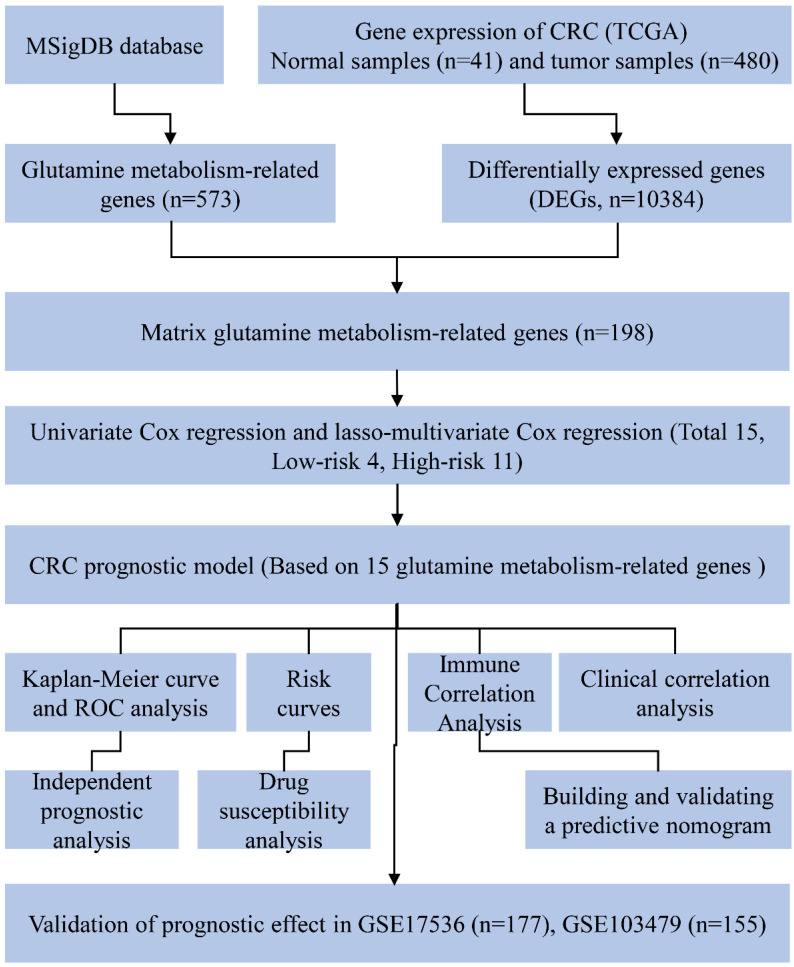
Experimental flowchart of the whole study.

**Figure 2:**
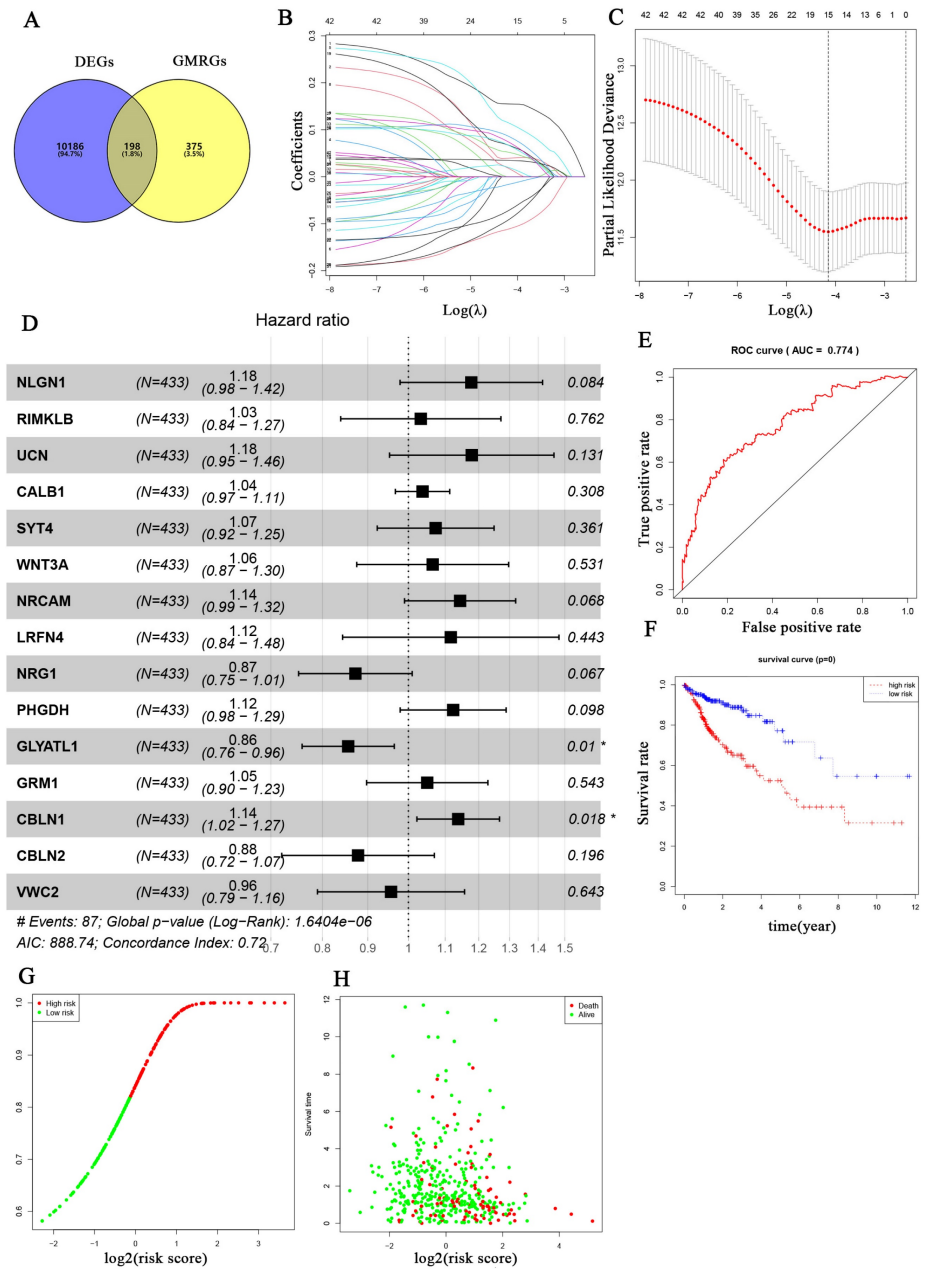
Development of the prognostic signature based on 15 glutamine metabolism related genes (GMRGs). (A) Intersection of DEGs with GMRGs. (B) The variation characteristics of the coefficient of variables; (C) The selection process of the optimum value of the parameter λ in the Lasso regression model by cross-validation method. (D) Assessment of GMRGs in predicting prognosis of CRC exhibited by forest plot. (E) ROC curve of the 15 GMRGs prognostic signature. (F) Overall survival (OS) of CRC patients in high- and low-risk groups. (G) Risk score distribution. (H) survival status in high- and low-risk groups.

The overall workflow of this study is depicted in Fig. 1. Firstly, a total of 573 genes related to glutamine metabolism were obtained from the MSigDB database. Secondly, 10,384 differentially expressed genes in CRC were retrieved from the TCGA database. Lastly, an intersection between glutamine metabolism-related genes and CRC differentially expressed genes was performed, resulting in a matrix of 198 genes associated with glutamine metabolism, as illustrated in Fig. 2A.

### Construction of a prognostic model associated with glutamine metabolism genes in CRC

After further screening the data of 480 CRC patients downloaded from the TCGA database, we found that 433 CRC patients had complete clinical information, including the survival data required for our study (Table 1). The patient demographics and baseline characteristics are presented in Table 2. To investigate the prognostic value of 198 glutamine metabolism-related genes in CRC, we initially conducted univariate Cox regression analyses. This analysis identified 42 genes that were significantly associated with the overall survival of CRC patients (p < 0.05). Further, we performed LASSO-multivariable Cox analysis on these 42 genes and determined that 15 glutamine metabolism-related genes were significantly associated with CRC prognosis (Table 2).

The regression coefficients in the LASSO regression model are displayed in Fig. 2B, while the optimal lambda values are shown in Fig. 2C. Additionally, we conducted multivariate COX regression analyses for the 15-glutamine metabolism-related genes, and identified GLYATL1 and CBLN1 as statistically significant (P < 0.05) (Fig. 2D). Among them, 11 genes (NLGN1, RIMKLB, UCN, CALB1, SYT4, WNT3A, NRCAM, LRFN4, PHGDH, GRM1, and CBLN1) were classified as high-risk genes (HR>1), and 4 genes (NRG1, GLYATL1, CBLN2, and VWC2) were classified as low-risk genes (HR<1). Lastly, we developed a risk assessment model for CRC based on these 15-glutamine metabolism-related genes and their corresponding coefficients obtained from multivariate Cox regression (Table 1). The formula for calculating the risk score is as follows: Risk score = (0.16316 * NLGN1) + (0.03212 * RIMKLB) + (0.16431 * UCN) + (0.03671 * CALB1) + (0.07064 * SYT4) + (0.06297 * WNT3A) + (0.13404 * NRCAM) + (0.10988 * LRFN4) + (-0.13748 * NRG1) + (0.11606 * PHGDH) + (-0.15622 * GLYATL1) + (0.04881 * GRM1) + (0.12913 * CBLN1) + (-0.13064 * CBLN2) + (-0.04510 * VWC2).

### Internal and external validation of prognostic model efficacy based on 15 glutamine metabolism-related genes

To assess the predictive ability of 15 genes related to glutamine metabolism for CRC, we conducted internal and external validation. In the internal validation, risk scores were calculated for 433 patients based on the risk coefficients. Subsequently, high-risk and low-risk groups were identified using the median risk scores. The two groups were then compared using time-dependent ROC curve and KM curve analyses. The area under the ROC curve (AUC) for risk score prediction was 0.774 (Fig. 2E), indicating that our prediction model, based on the 15 glutamine metabolism genes, has excellent predictive ability for CRC. KM curve analysis (Fig. 2F) demonstrated that overall survival (OS) was worse in the high-risk group compared to the low-risk group (p < 0.0001). Additionally, the distribution of risk scores and survival time in the two groups showed that patients in the low-risk group had longer survival compared to those in the high-risk group (Fig. 2G-H).

**Figure 3:**
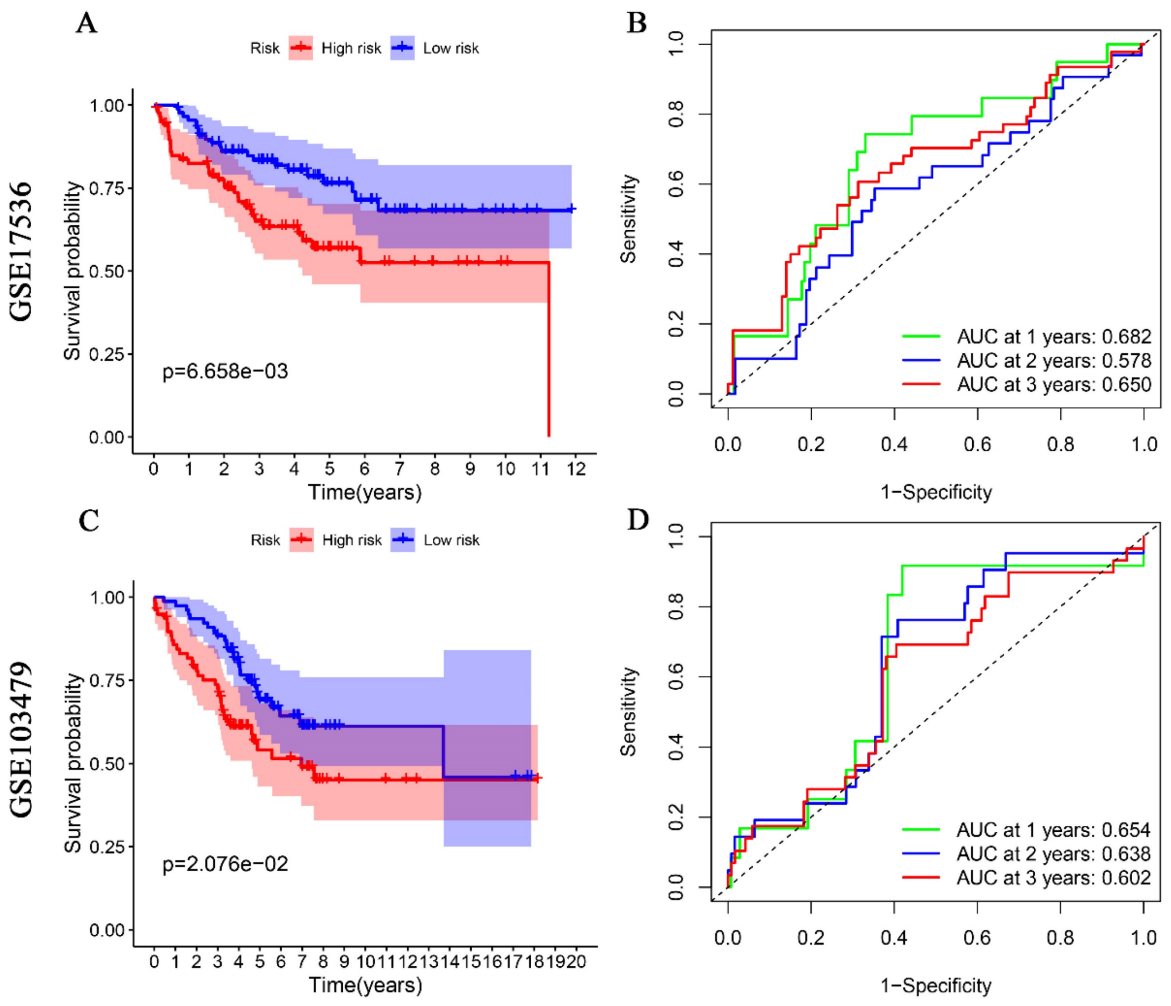
Validation of glutamine metabolism-related genes associated with overall survival in colorectal cancer in GSE17536 and GSE103479 datasets. Kaplan-Meier analyses suggest that CRC patients with high-risk scores have a worse overall prognosis (A, and C). ROC analyses of risk scores assessed sensitivity and specificity (B, and D).

For external validation, two datasets from the GEO database (GSE17536, GSE103479) were used as a test set to validate the prognostic value of glutamine metabolism-associated genetic risk profiles in CRC. Similarly, CRC patients in these datasets were divided into high-risk and low-risk groups for Kaplan-Meier analysis and ROC analysis. Kaplan-Meier analysis indicated that CRC patients in the high-risk group had a worse overall prognosis than those in the low-risk group (Fig. 3A, 3C). ROC analysis results showed that the AUCs for 1, 2, and 3 years were 0.682, 0.578, and 0.650 in the GSE17536 dataset (Fig. 3B), and 0.654, 0.638, and 0.602 in the GSE103479 dataset, respectively (Fig. 3D). The above results indicate that the prediction model we constructed has good predictive ability for the survival of CRC patients.

### Correlation of glutamine metabolism-related prediction models with overall survival in CRC patients

**Figure 4:**
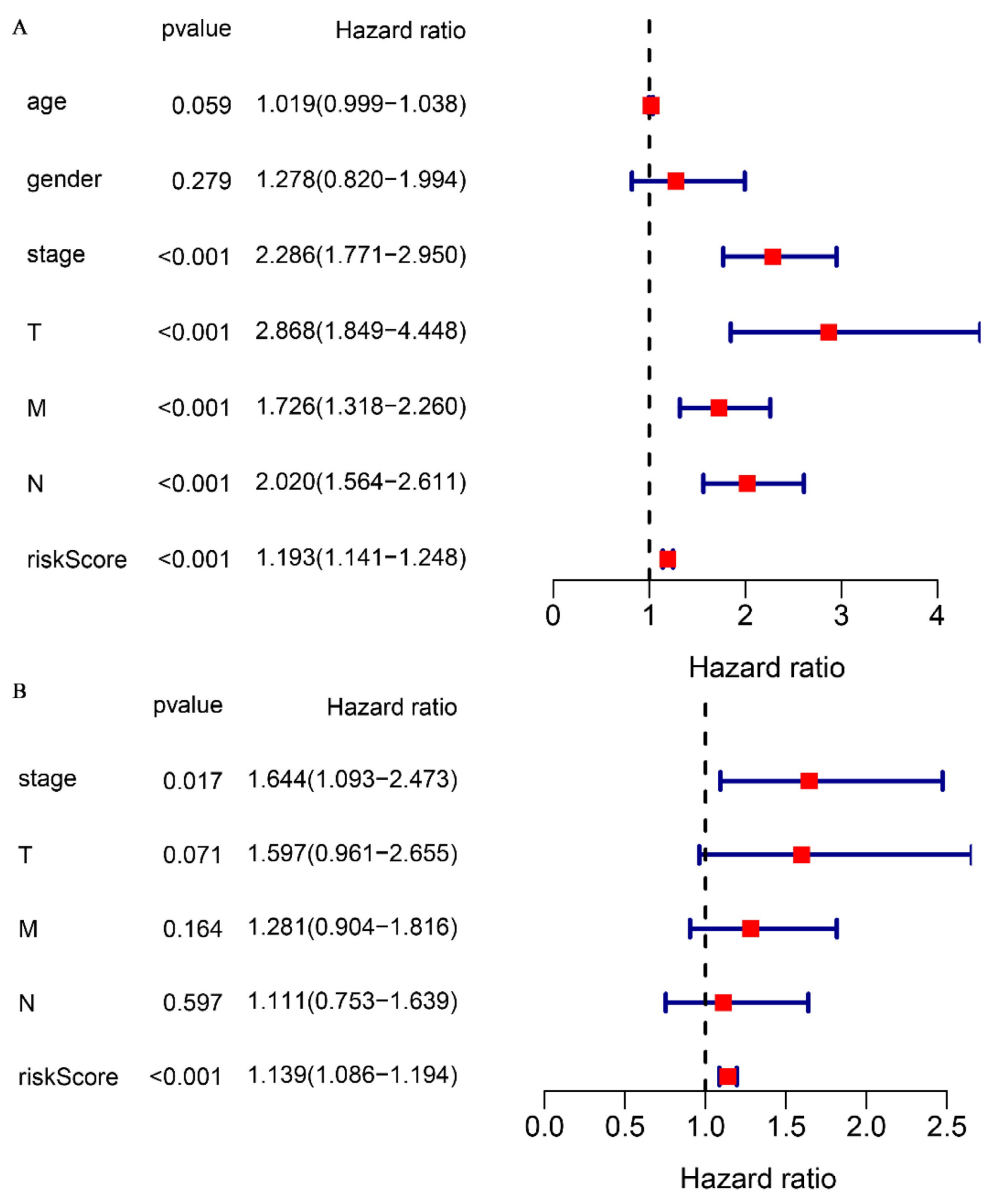
Association of glutamine metabolism-related characteristics and clinical factors with overall survival in the TCGA dataset. Univariate Cox regression analyses (A) and multivariate Cox regression analyses (B) of glutamine metabolism-related characteristics and clinical factors with overall survival.

To validate the correlation between the prognostic model and overall survival in patients, a univariate COX regression analysis was performed. The analysis demonstrated that clinical stage (P < 0.001), TNM stage (P < 0.001), and risk score (P < 0.001) were significantly correlated with overall survival (Fig. 4A). Multifactorial COX analysis revealed that clinical stage (P < 0.05) and risk score (P < 0.001) remained as independent prognostic factors associated with overall survival in CRC patients (Fig. 4B). In summary, our prognostic model for glutamine metabolism is an independent and reliable factor for assessing CRC prognosis.

### Association between glutamine metabolism-related prediction models and clinical features

**Figure 5:**
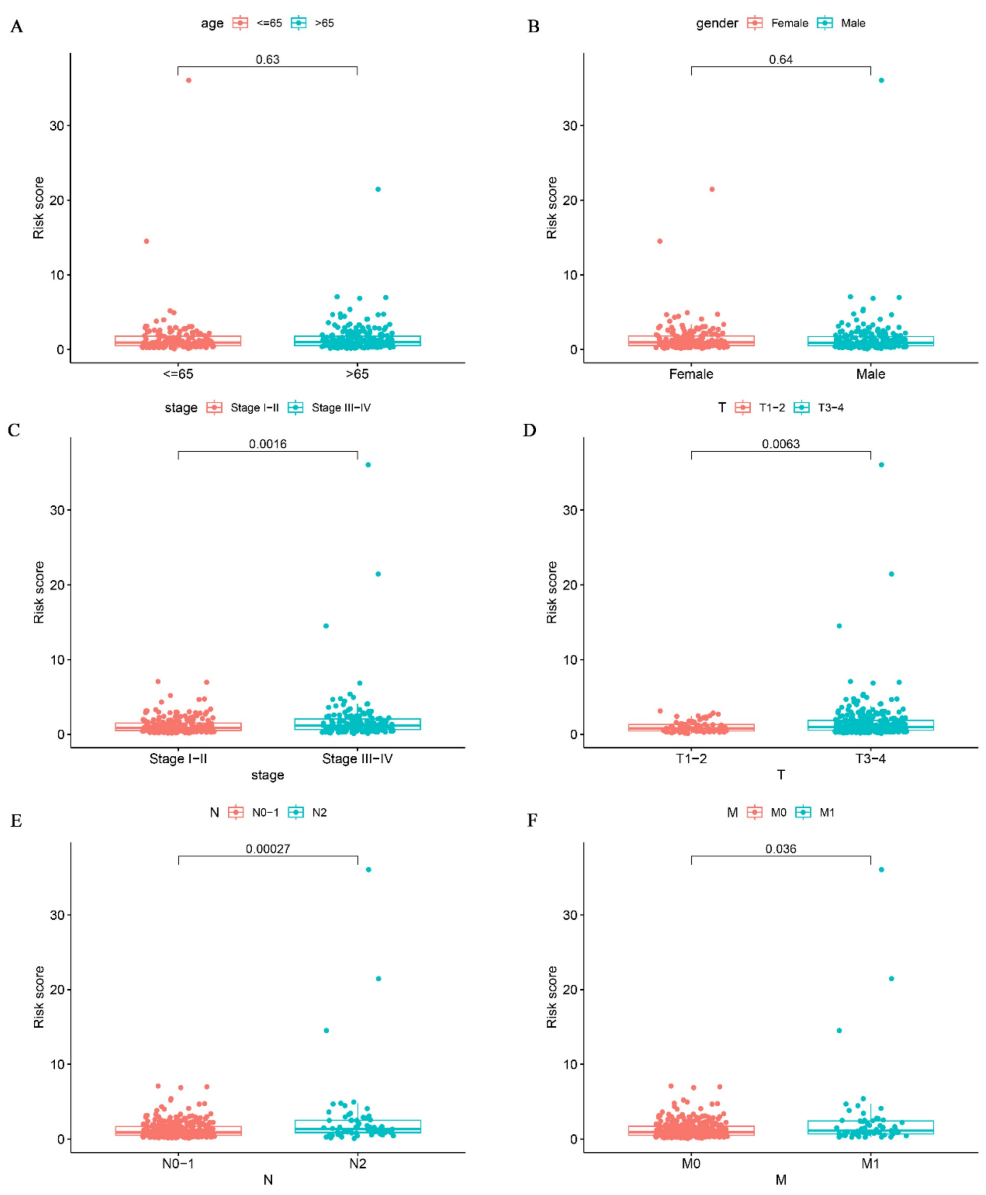
Correlation analysis of glutamine metabolism-related characteristics with clinical variables. Association of risk scores with age (A), gender (B), stage (C), T classification (D), and N classification (E) and M classification (F).

To investigate the relationship between risk scores and clinical signatures, we analyzed the prognostic model for correlation with patient clinical parameters in 433 patients of CRC. The results showed that risk scores were higher in patients with more advanced clinical staging and T, N, and M staging than in patients with earlier staging (Fig. 5C-F), which demonstrated that the prognostic model was reliable. Regrettably, there was no statistically significant difference in risk scores between age and gender as shown in Fig. 5A-B (p > 0.05).

### Glutamine metabolism-related prediction models with immune correlation analysis

**Figure 6:**
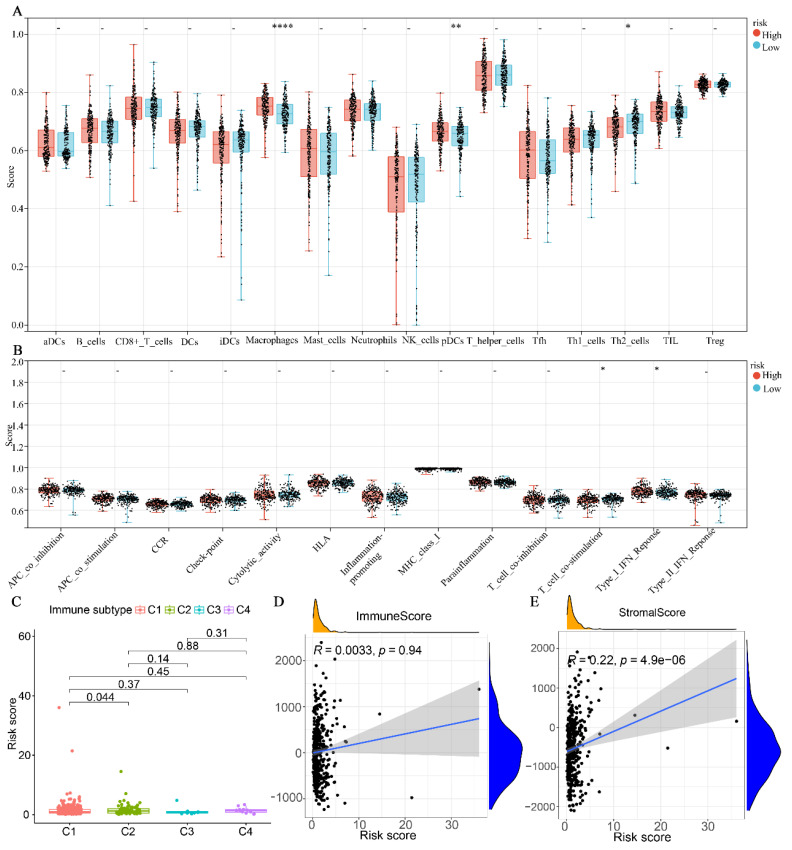
Association of glutamine metabolism-related characteristics with immune cells in the TCGA dataset. (A) Differential fractions of immune cells in low- and high-risk groups. (B) Different immune cell functions in low- and high-risk groups. (C) Relationship between immune subtype and risk score. (D) Correlation analysis between immune score and risk score. (E) Correlation analysis between stromal score and risk score.

The results indicated that the high-risk group exhibited significantly higher levels of Macrophages and plasmacytoid dendritic cells (pDCs) compared to the low-risk group. Conversely, the high-risk group demonstrated lower levels of Th2 cells compared to the low-risk group (Fig. 6A). Regarding immune cell function, the high-risk group was primarily associated with T-cell co-stimulation, whereas the low-risk group was predominantly associated with type I interferon (INF) responses (Fig. 6B). In terms of immune subtypes, the risk score for C2 was found to be higher than that of C1 (P < 0.05). However, no significant difference was observed between C3 and C4 (Fig. 6C). Additionally, we further analyzed the correlation between the immunity score and the stromal score with the risk score. As depicted in Fig. 6D-E, the stromal score exhibited a significant positive correlation with the risk score (P < 0.0001). However, no significant correlation was observed between the immune score and the risk score (P = 0.94).

### Correlation analysis of genes related to glutamine metabolism with drug sensitivity

**Figure 7:**
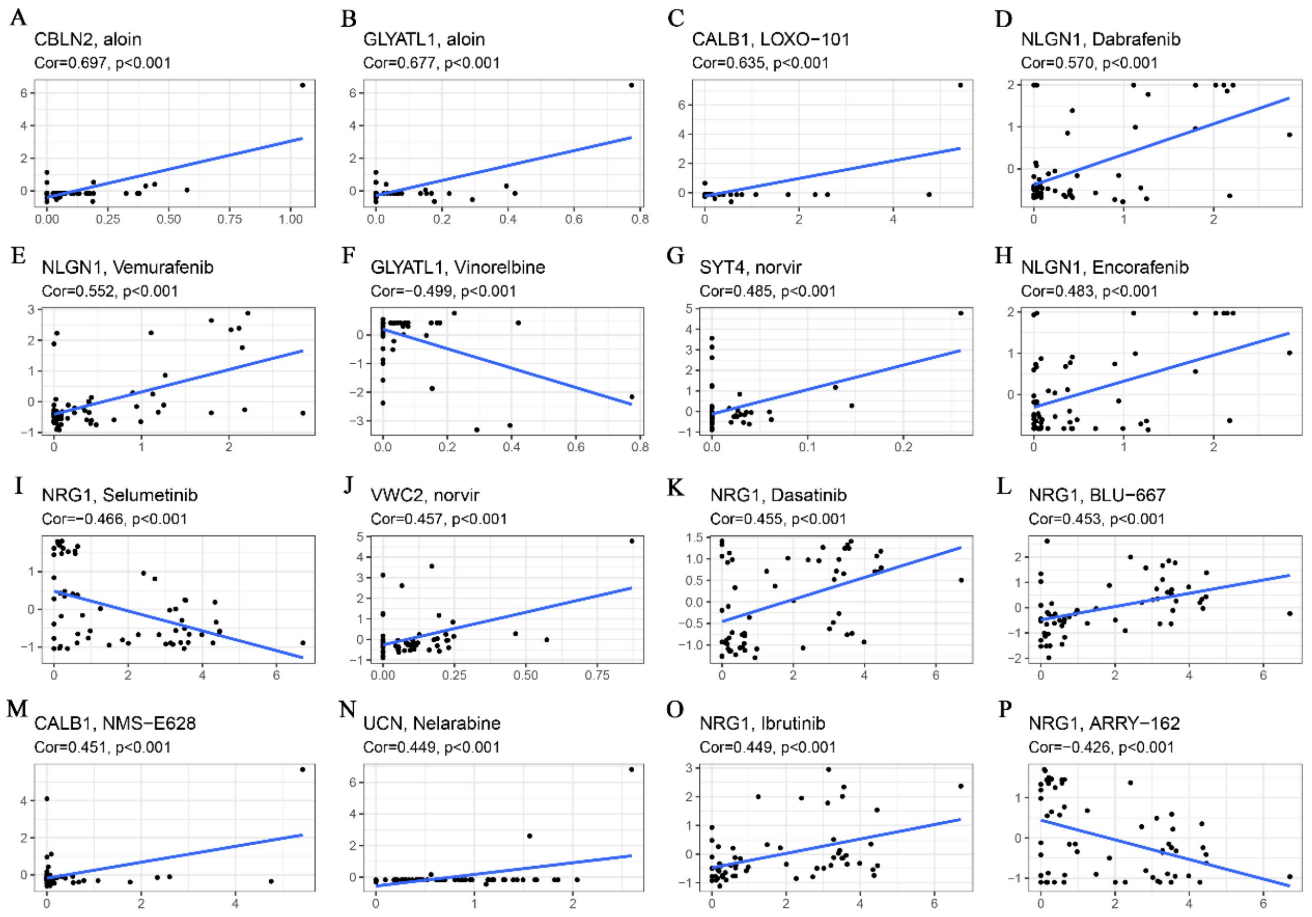
Correlation analysis of glutamine metabolism-related characteristics with drug sensitivity. (A) CBLN2 was positively correlated with aloin sensitivity. (B) GLYATL1 was positively correlated with aloin sensitivity. (C) CALB1 was positively correlated with LOXO-101 sensitivity. (D) NLGN1 was positively correlated with dabrafenib sensitivity. (E) NLGN1 was positively correlated with vemurafenib sensitivity. (F) GLYATL1 was negatively correlated with vinorelbine sensitivity. (G) SYT4 was positively correlated with norvir sensitivity. (H) NLGN1 was positively correlated with encorafenib sensitivity. (I) NRG1 was negatively correlated with selumetinib sensitivity. (J) VWC2 was positively correlated with norvir sensitivity. (K) NRG1 was positively correlated with dasatinib sensitivity. (L) NRG1 was positively correlated with BLU-667 sensitivity. (M) CALB1 was positively correlated with NMS-E628 sensitivity. (N) UCN was positively correlated with Nelarabine sensitivity. (O) NRG1 was positively correlated with Ibrutinib sensitivity. (P) NRG1was negatively correlated with ARRY-162 sensitivity.

Drug sensitivity analyses revealed that both CBLN2 and GCYATL1 expression were positively correlated with aloin sensitivity (Fig. 7A-B) (p < 0.001), while GCYATL1 was negatively correlated with Vinorelbine sensitivity (Fig. 7F) (p < 0.001). Additionally, the drug sensitivity of LOXO-101 (Fig. 7C) and NMS-E628 (Fig. 7M) was enhanced with elevated expression levels of CABL1 (p < 0.001). Similarly, the drug sensitivity of Dabrafenib (Fig. 7D), Vemurafenib (Fig. 7E), and Encorafenib (Fig. 7H) showed a strong positive correlation with NLGN1 expression (p < 0.001). As depicted in Fig. 7G and Fig. 7J, higher expression levels of SYT4 and VWC2 were associated with stronger drug sensitivity to norvir (p < 0.001). An intriguing observation was that NRG1 expression demonstrated a significant positive correlation (p < 0.001) with the sensitivity of drugs such as Dasatinib (Fig. 7K), BLU-667 (Fig.7L), and Ibrutinib (Fig.7O), while exhibiting a highly significant negative correlation (p < 0.001) with the sensitivity of Selumetinib (Fig. 7I) and ARRY-162 (Fig. 7P). Finally, Fig. 7N indicates that higher expression of UCN was associated with greater drug sensitivity to Nelarabine (p < 0.001).

### Construction of the Nomogram Model

**Figure 8:**
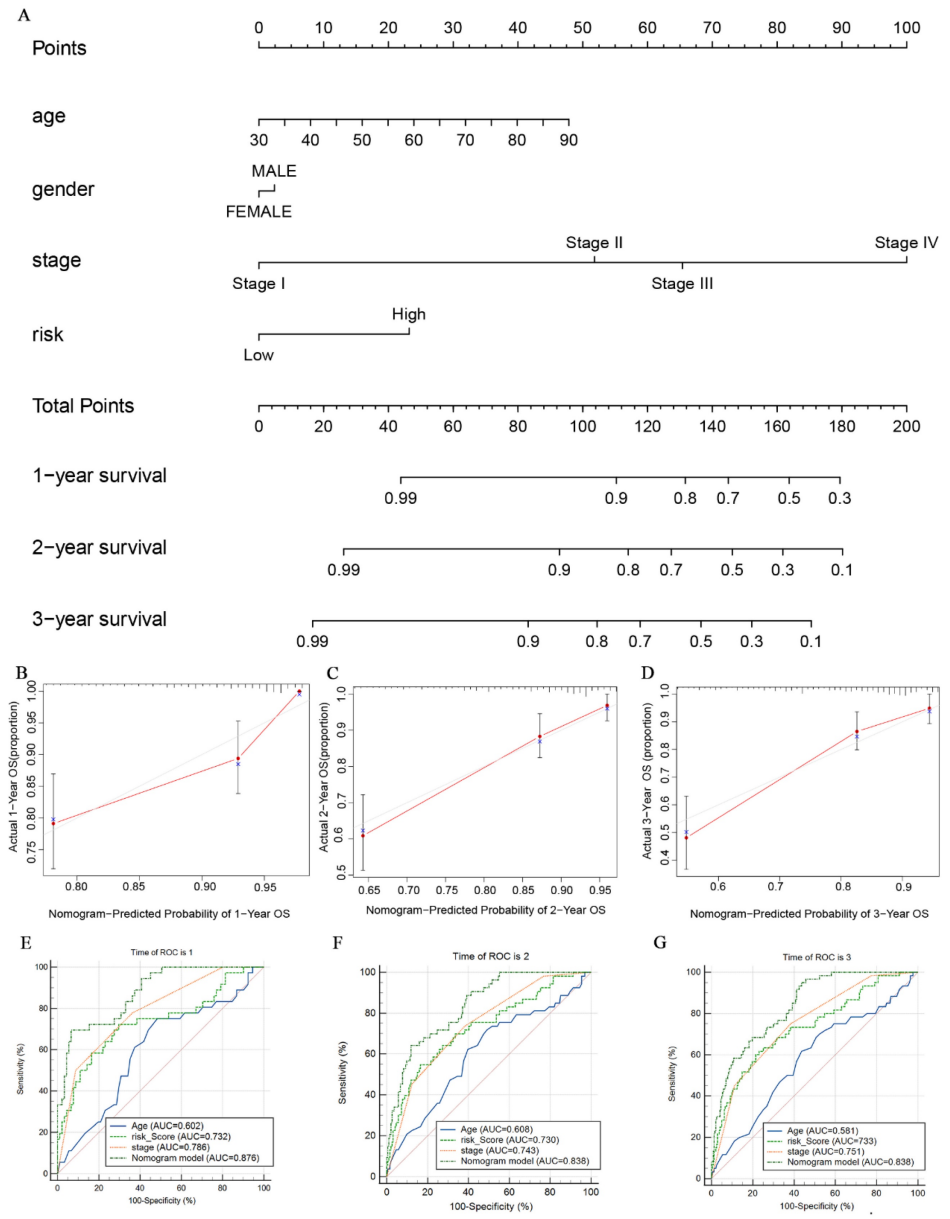
The nomogram constructed to predict overall survival (OS) in the clinical setting presents good prediction ability. (A) A nomogram created by the combination of the risk score and TNM stage to predict the OS of CRC. (B-D) Calibration charts predicting 1-, 2- and 3-year survival in TCGA dataset. The horizontal axis and vertical axis represent the predicted survival probability and the actual survival probability. (E-G) ROC curve of the clinical prognostic nomogram for predicting 1-, 2-, and 3-year survival in CRC patients.

To better assess the probabilities of 1-, 2-, and 3-year survival in CRC patients, we developed a nomogram model. This model integrates the scores of clinical features such as age, gender, clinical stage, and glutamine metabolism-related gene features (Fig. 8A). The nomogram model allows us to observe that the survival probability of CRC patients in the low-risk group is higher than that of those in the high-risk group. Additionally, the calibration curves demonstrate that the nomogram effectively predicts 1-, 2- and 3-year overall survival (OS) in line with the actual OS (Fig. 8B-D). As shown in Fig. 8E-G, the nomogram AUC values for 1-, 2-, and 3-year OS were 0.876, 0.838, and 0.838, respectively. This further suggests that the glutamine metabolism-related gene profile exhibits excellent predictive ability for CRC patients.

### Verification of the expression levels of glutamine metabolism-related core genes in CRC samples

**Figure 9:**
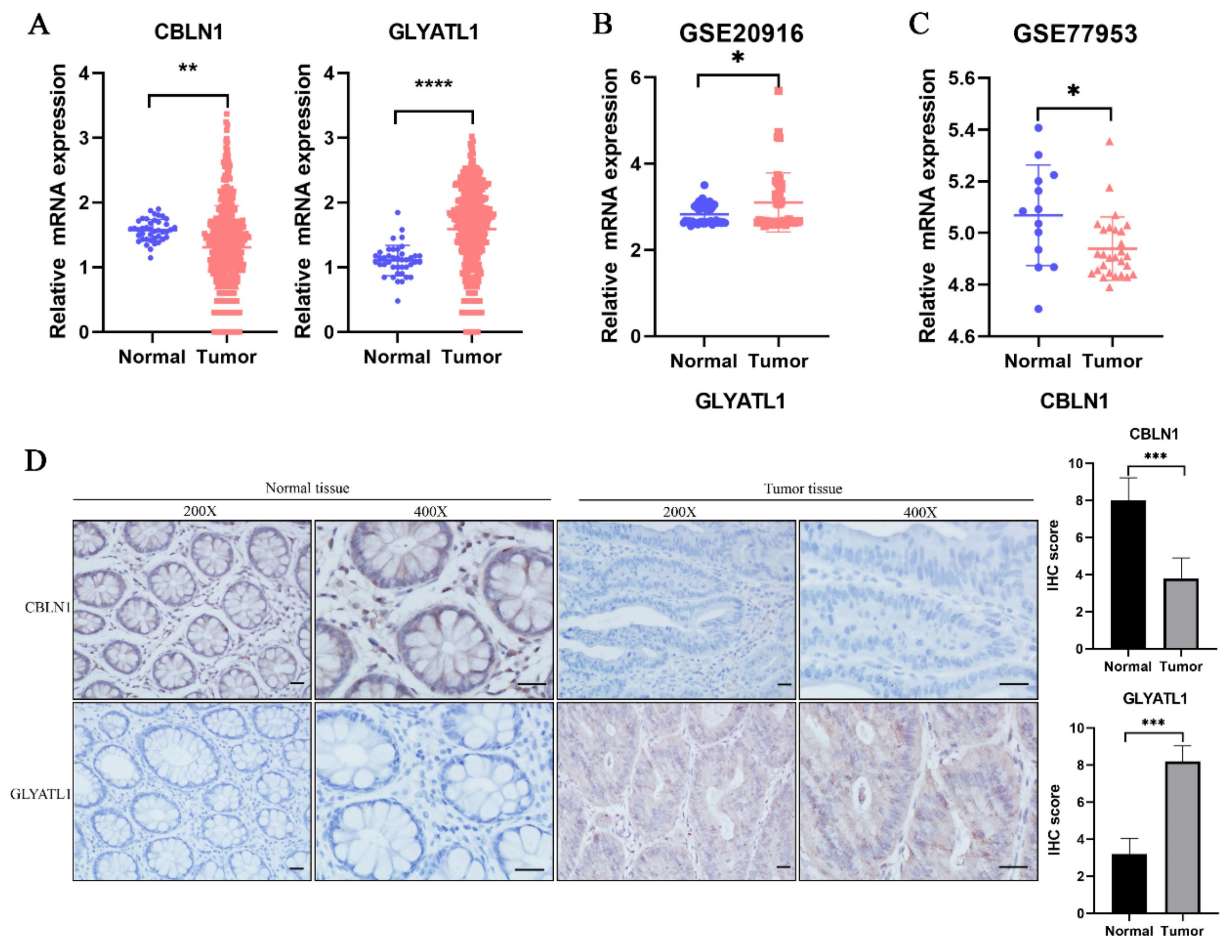
Expression analysis of CBLN1 and GLYATL1. (A) The expression of CBLN1 and GLYATL1 in the normal and tumor tissues based on the TCGA dataset. (B) Validation of the expression of GLYATL1 in tumor and normal tissues in GSE20916 dataset. (C) Validation of the expression of CBLN1 in tumor and normal tissues in GSE77953 dataset. (D) The immunohistochemical staining results showed significant differences of CBLN1 and GLYATL1 at the protein expression between normal and tumor tissues.

In the previous analysis, as shown in Fig. 2D, we found that among the 15-glutamine metabolism-related genes, CBLN1 and GLYATL1 were independent factors for CRC prognosis according to multifactorial COX regression (P < 0.05). To further assess the expression levels of the genes CBLN1 and GLYATL1, which are important for the construction of CRC prognostic features, we first predicted their expression by downloading from the TGGA database and GEO database (Fig. 9A-C). We observed that CBLN1 was expressed at a low level in normal tissues compared to CRC tissues, while GLYATL1 was highly expressed. Additionally, we performed immunohistochemical (IHC) experiments to assess the expression levels of CBLN1 and GLYATL1 in 10 collected CRC tissues and adjacent normal tissues. As shown in Fig. 9D, CBLN1 was expressed at a low level in CRC tissues compared to normal tissues, whereas GLYATL1 was highly expressed in CRC tissues, consistent with the results from the database.

## Discussion

CRC is one of the most common malignant tumors. It has been reported that there were over 1.9 million new cases of CRC and approximately 940,000 deaths among CRC patients worldwide in 2020, with an estimated increase to 3.2 million new cases by 2040 27. Despite improvements in the overall survival rate of CRC patients over the past 30 years 28, the 5-year survival rate for CRC remains unsatisfactory, particularly for patients with late-stage CRC and distant metastases 29-31. Therefore, it is crucial to develop a new prognostic model for assessing the prognosis of CRC patients, which would guide clinicians in early intervention for those at high risk. Several previous studies have highlighted the close relationship between glutamine metabolism and the development of CRC 32-34. For instance, PIK3CA mutations have been found to increase glutamine dependence in CRC cells, suggesting that inhibiting glutamine metabolism could be a potential therapeutic approach for CRC 18. However, only a limited number of genes related to glutamine metabolism have been extensively studied in CRC. This study is the first to investigate the prognostic value of glutamine metabolism-related genes in CRC, offering new insights into the diagnosis, treatment, and prevention of CRC.

In this study, we identified a total of 198 genes related to glutamine metabolism in colorectal cancer (CRC) from the MSigDB and TCCA databases. Subsequently, the 198 genes underwent Univariate Cox regression and lasso-multivariate Cox regression analyses, leading us to select 15 genes associated with glutamine metabolism for the development of prognostic models for CRC. Out of these 15 genes, 11 (NLGN1, RIMKLB, UCN, CALB1, SYT4, WNT3A, NRCAM, LRFN4, PHGDH, GRM1, and CBLN1) were identified as high-risk genes, while 4 genes (NRG1, GLYATL1, CBLN2, and VWC2) were classified as low-risk genes.

The majority of these 15 key genes have been reported to be closely associated with the development and prognosis of CRC or other malignant tumors. Reportedly, NLGN1 facilitates CRC development by mediating the APC/β-catenin pathway 35. Most of these 15 key genes have previously been implicated in the development and prognosis of CRC and other malignant tumors. For example, NLGN1 has been reported to facilitate CRC development by mediating the APC/β-catenin pathway 32. Additionally, studies have shown that upregulation of NLGN1, RIMKLB, and CALB1 is associated with poor prognosis in CRC patients 36-38. WNT signaling pathway is known to play a crucial role in the development of many cancers, and WNT3A, a classical WNT ligand, has been shown to activate the WNT/β-collagen pathway, thereby promoting CRC progression 39, 40. NRCAM, a gene encoding a neuronal cell adhesion molecule, promotes CRC through the beta-catenin/LEF-1 pathway 41. It has also been demonstrated that NRCAM, LRFN4, and PHGDH are upregulated in CRC tissues compared to normal samples around the tumor and serve as predictors of poor clinical outcomes in advanced CRC patients 42-44. Tumor genome sequencing data have indicated that GRM1 is one of the pivotal driving genes for CRC stage II progression 45. NRG1 gene fusions have been identified as oncogenic drivers in various solid tumors, including CRC, gallbladder, pancreatic, and bladder cancers 46. Low expression of GLYATL1 can serve as a predictor of poor prognosis in patients with clear cell renal carcinoma and hepatocellular carcinoma 47, 48. Moreover, overexpression of VWC2 has been shown to inhibit the proliferation of HCT-116 and HT29 cells 49. Although the roles of genes such as UCN, SYT4, CBLN1, CBLN2, and GLYATL1 in CRC remain unclear, our findings suggest that they can still serve as potential biomarkers for CRC prognosis.

To further validate the efficacy of the glutamine metabolism-related model, we conducted internal and external validation. A total of 433 CRC samples with complete clinical information were divided into high-risk and low-risk groups based on the median risk score. In the internal validation, the ROC analysis suggested that the model had an AUC of 0.74, indicating excellent sensitivity and specificity. Kaplan-Meier survival analyses revealed that the high-risk group of CRC patients had a significantly lower survival rate compared to the low-risk group of CRC patients. External validation of the model using the GSE17536 and GSE103479 datasets similarly confirmed that the overall survival of CRC patients in the low-risk group was higher than in the high-risk group. These results demonstrate the stability of using glutamine metabolism-related genes for assessing the prognosis of CRC patients. Univariate and multivariate COX regressions showed that the risk score was an independent factor in the prognosis of CRC, further confirming the reliability of this predictive model. In terms of clinical relevance, the risk score was significantly correlated with clinical stage and TNM stage, suggesting a potentially higher risk score in patients with advanced CRC.

In recent years, there has been increasing evidence linking immune cell infiltration with the development and progression of CRC, suggesting it can serve as a prognostic marker 50, 51. To comprehensively explore the correlation between risk scores and immune cell infiltration, we utilized the ssGSEA algorithm to compare the variability of immune cells in the high- and low-risk groups. Our findings revealed that the high-risk group had a predominant enrichment of macrophages and pDCs, while the low-risk group had a predominant enrichment of Th2 cells. It has been reported that high infiltration of macrophages exacerbates the progression of ulcerative colitis to colon cancer and is considered a poor prognostic marker 52, 53. Similarly, extensive infiltration of pDCs predicts a poor prognosis in breast and ovarian cancer 54, 55. Conversely, increasing Th2 cell infiltration is considered a marker of good prognosis in CRC 56. In our study, we observed a higher frequency of T-cell co-stimulation in the high-risk group. Enhanced T-cell co-stimulation is often observed to generate a stronger immune response against tumors, which may explain its increased occurrence in the high-risk group 57. Interestingly, we found that type I interferon responses were more likely to occur in the low-risk group. This may be attributed to the fact that type I interferon responses enhance T-cell responses, leading to anti-tumor effects 58. Moreover, we observed a positive correlation between the risk score and the stromal score, which is consistent with previous reports indicating that a low stromal score is protective against CRC 59. Furthermore, our analysis suggests that glutamine metabolism-related genes are closely associated with immune cell infiltration, and the poor prognosis observed in high-risk CRC patients may be the result of a disturbed tumor immune microenvironment. To explore the correlation between glutamine metabolism-related genes and antitumor drug sensitivity, we utilized the CellMiner database. We found that most glutamine metabolism-related genes demonstrated a positive correlation with drug sensitivity. For instance, higher expression levels of CBLN2 and GCYATL1 were associated with increased sensitivity to aloin drugs. Aloin has been reported to inhibit the proliferation and induce apoptosis of CRC cells, making it a potential candidate for CRC treatment 60.

In our study, the training cohort and two validation cohorts (GSE17536, GSE103479) predicted 1-year OS with AUC values of 0.733, 0.682, and 0.654, which were higher than those with other similar prognostic features (AUC=0.585) 61, suggesting that our developed prediction model performed better. As part of our study, we also developed a nomogram model that is based on age, gender, clinical stage, and risk score. The results of our study have confirmed that the nomogram exhibits good stability and reliability in predicting the 1-, 2-, and 3-year OS in CRC patients. We developed nomograms predicting 1- and 3-year OS with AUC values of 0.876,0.838, which were higher than other similar prognostic features previously developed 62-64. Additionally, we collected tissue specimens from CRC patients, as well as adjacent normal tissue specimens, from a total of 10 surgical patients. IHC was used to verify the expression levels of the two most significant genes, CBLN1 and GLYATL1, in a glutamine metabolism-related profile. Our findings indicate that CBLN1 expression was lower and GLYATL1 expression was higher in CRC tissues compared to normal tissues, which is consistent with the predictions made in the TCCA and GEO databases. However, it should be noted that our study is retrospective in nature and is based on a database. In order to enhance the credibility of our findings, further multi-centric and prospective studies need to be conducted. Additionally, the mechanism of action of glutamine metabolism-related genes in CRC requires further validation through *in vivo* and *in vitro* studies.

## Conclusion

In conclusion, we have successfully identified and comprehensively analyzed a gene signature related to glutamine metabolism. This signature has shown excellent reliability and stability in predicting the prognosis of CRC patients. The findings from this study systematically highlight the role of glutamine metabolism-related genes in CRC and provide new insights for the clinical diagnosis, treatment, and prevention of CRC.

## Figures and Tables

**Table 1:** Patient demographics and baseline characteristics

Characteristic	Risk level		p-value^2^
	High, N = 216^1^	Low, N = 217^1^	
**Age**			0.586
Mean ± SD	66 ± 13	67 ± 12	
**Gender**			0.812
MALE	115 (53%)	118 (54%)	
FEMALE	101 (47%)	99 (46%)	
**Stage**			0.005
Stage I	27 (13%)	46 (21%)	
Stage II	75 (35%)	90 (41%)	
Stage III	76 (35%)	47 (22%)	
Stage IV	34 (16%)	27 (12%)	
unknow	4 (2%)	7 (3%)	
**T**			0.002
Tis	0 (0%)	1 (0%)	
T1	3 (1%)	8 (4%)	
T2	29 (13%)	46 (21%)	
T3	149 (69%)	147 (68%)	
T4	35 (16%)	15 (7%)	
**M**			0.405
M0	155 (72%)	165 (76%)	
M1	34 (16%)	27 (12%)	
MX	25 (12%)	20 (9%)	
unknown	2 (1%)	5 (2%)	
**N**			<0.001
N0	108 (50%)	146 (67%)	
N1	52 (24%)	50 (23%)	
N2	56 (26%)	21 (10%)	
**State of survival**			<0.001
Alive	154 (71%)	192 (88%)	
Death	62 (29%)	25 (12%)	

^1^n (%); ^2^Welch Two Sample t-test; Pearson's Chi-squared test; Fisher's exact test

**Table 2:** Coefficients and lasso-multivariable Cox model results of 15 glutamine metabolism-related genes in the prognostic model of CRC.

	coef	exp(coef)	se(coef)	z	Pr(>|z|)
NLGN1	0.16316	1.17723	0.09429	1.730	0.0836
RIMKLB	0.03212	1.03265	0.10602	0.303	0.7619
UCN	0.16431	1.17858	0.10880	1.510	0.1310
CALB1	0.03671	1.03739	0.03599	1.020	0.3077
SYT4	0.07064	1.07320	0.07732	0.914	0.3609
WNT3A	0.06297	1.06499	0.10051	0.626	0.5310
NRCAM	0.13404	1.14343	0.07355	1.822	0.0684
LRFN4	0.10988	1.11615	0.14315	0.768	0.4427
NRG1	-0.13748	0.87155	0.07515	-1.829	0.0673
PHGDH	0.11606	1.12307	0.07012	1.655	0.0979
GLYATL1	-0.15622	0.85537	0.06102	-2.560	0.0105*
GRM1	0.04881	1.05002	0.08026	0.608	0.5431
CBLN1	0.12913	1.13784	0.05466	2.363	0.0182*
CBLN2	-0.13064	0.87753	0.10100	-1.293	0.1958
VWC2	-0.04510	0.95591	0.09734	-0.463	0.6432
